# Construction of a synthetic infectious cDNA clone of *Grapevine Algerian latent virus* (GALV-Nf) and its biological activity in *Nicotiana benthamiana* and grapevine plants

**DOI:** 10.1186/1743-422X-11-186

**Published:** 2014-11-03

**Authors:** Arianna Lovato, Franco Faoro, Giorgio Gambino, Dario Maffi, Marcella Bracale, Annalisa Polverari, Luca Santi

**Affiliations:** Department of Biotechnology, University of Verona, Strada le Grazie 15, 37134 Verona, Italy; Department of Agricultural and Environmental Sciences (DiSAA), University of Milan, via Celoria 2, 20133 Milan, Italy; Institute for Sustainable Plant Protection, National Research Council, Grugliasco Unit, Largo Braccini 2, 10095 Grugliasco (TO), Italy; Department of Environment, Health and Safety, University of Insubria, via J.H. Dunant 3, 21100 Varese, Italy; Department of Science and Technology for Agriculture, Forestry, Nature and Energy (DAFNE), University of Tuscia, Via San Camillo de Lellis snc, 01100 Viterbo, Italy

**Keywords:** Plant viruses, Synthetic biology, Grapevine, GALV, Cytopathology

## Abstract

**Background:**

*Grapevine Algerian latent virus* (GALV) is a tombusvirus first isolated in 1989 from an Algerian grapevine (*Vitis* spp.) plant and more recently from water samples and commercial nipplefruit and statice plants. No further reports of natural GALV infections in grapevine have been published in the last two decades, and artificial inoculations of grapevine plants have not been reported. We developed and tested a synthetic GALV construct for the inoculation of *Nicotiana benthamiana* plants and different grapevine genotypes to investigate the ability of this virus to infect and spread systemically in different hosts.

**Methods:**

We carried out a phylogenetic analysis of all known GALV sequences and an epidemiological survey of grapevine samples to detect the virus. A GALV-Nf clone under the control of the T7 promoter was chemically synthesized based on the full-length sequence of the nipplefruit isolate GALV-Nf, the only available sequence at the time the project was conceived, and the infectious transcripts were tested in *N. benthamiana* plants. A GALV-Nf-based binary vector was then developed for the agroinoculation of *N. benthamiana* and grapevine plants. Infections were confirmed by serological and molecular analysis and the resulting ultrastructural changes were investigated in both species.

**Results:**

Sequence analysis showed that the GALV coat protein is highly conserved among diverse isolates. The first epidemiological survey of cDNAs collected from 152 grapevine plants with virus-like symptoms did not reveal the presence of GALV in any of the samples. The agroinoculation of *N. benthamiana* and grapevine plants with the GALV-Nf binary vector promoted efficient infections, as revealed by serological and molecular analysis. The GALV-Nf infection of grapevine plants was characterized in more detail by inoculating different cultivars, revealing distinct patterns of symptom development. Ultrastructural changes induced by GALV-Nf in *N. benthamiana* were similar to those induced by tombusviruses in other hosts, but the cytopathological alterations in grapevine plants were less severe.

**Conclusions:**

This is the first report describing the development of a synthetic GALV-Nf cDNA clone, its artificial transmission to grapevine plants and the resulting symptoms and cytopathological alterations.

**Electronic supplementary material:**

The online version of this article (doi:10.1186/1743-422X-11-186) contains supplementary material, which is available to authorized users.

## Background

*Grapevine Algerian latent virus* (GALV) was first isolated in Italy in 1989 from an Algerian vine infected by *Grapevine fanleaf virus* (GFLV) [1]. GALV was considered a latent virus because the infected plant showed only GFLV-related symptoms, and there have been no further reports describing the detection of GALV in grapevine plants in the last 25 years. GALV has subsequently been isolated from waterways in western Sicily [2], from ditches and streams in agricultural areas of Germany [3], from water samples and different wild plants [4], and also from rivers and *Gypsophila paniculata* [5]. GALV has also been isolated from Dutch samples of commercially-grown statice plants and the groundwater of a statice production glasshouse [5], in Japan from commercially-cultivated nipplefruit [6] and *Limonium sinuatum* [7], and in Korea from *Limonium sinense* [8]. The potential economic consequences of this virus in the grapevine industry are unknown because it has been detected only once in a mixed infection [1] but it caused severe stunting, chlorotic spots and mosaic symptoms in nipplefruit and statice plants [6, 7]. Several experimental hosts have been identified in plant families such as *Solanaceae*, *Chenopodiaceae* and *Amaranthaceae* [1–3, 5–7].

GALV was assigned to the genus *Tombusvirus* in the family *Tombusviridae* based on its biological and physicochemical properties [1, 3]. The type member of this family is *Tomato bushy stunt virus* (TBSV), which has been studied in detail to characterize the molecular biology of the tombusviruses [9]. TBSV has been used to develop expression vectors [10, 11] and gene silencing approaches [12, 13]. The GALV genome is a positive single-stranded RNA ~4730 nucleotides in length, comprising a 5′ untranslated region (UTR), at least five open reading frames (ORFs) [6, 14] and a 3′ UTR. The p33 and p92 ORFs encode the replicase proteins which generate two major subgenomic RNAs, one for the p40 coat protein (CP) and the other containing two nested ORFs for the production of the p24 movement protein (MP) and the p19 multifunctional protein, which also functions as a silencing suppressor [15]. GALV is assembled into 30-nm icosahedral particles which accumulate in the cytoplasm. Infection results in the formation of specific membrane-associated vesicles at the periphery of peroxisomes in *Gomphrena globosa* [1]. Furthermore, peripheral vesiculation of mitochondria and chloroplasts [1] and dark staining rod-like structures in the cytoplasm and in the stroma of mitochondria and chloroplasts [3] were observed in *Chenopodium quinoa*.

There have been no reports of *Agrobacterium*-mediated GALV infection and attempts to inoculate grapevine plants with natural isolates of the virus have thus far failed [1, 6]. We therefore constructed synthetic GALV vectors based on the available genome sequence of the GALV isolate from nipplefruit (GALV-Nf [6]). These vectors were used to inoculate *N. benthamiana* and grapevine plants, which allowed us to investigate the ability of synthetic GALV-Nf constructs to replicate, spread systemically and assemble into normal virus particles. We characterized the resulting infections in detail, describing the development of symptoms and the cytopathological features of infection in both species.

## Results

### Variability of GALV isolates

We collected all available published GALV sequences by searching the NCBI Nucleotide Database, which yielded a single complete genome sequence derived from a nipplefruit isolate (GALV-Nf) plus seven further GALV coat protein mRNA sequences. A further complete genome sequence derived from a grapevine isolate [1] was also deposited recently, which hereafter we describe as GALV-Vv.2 to distinguish it from the GALV-Nf sequence.

**Figure 1:**
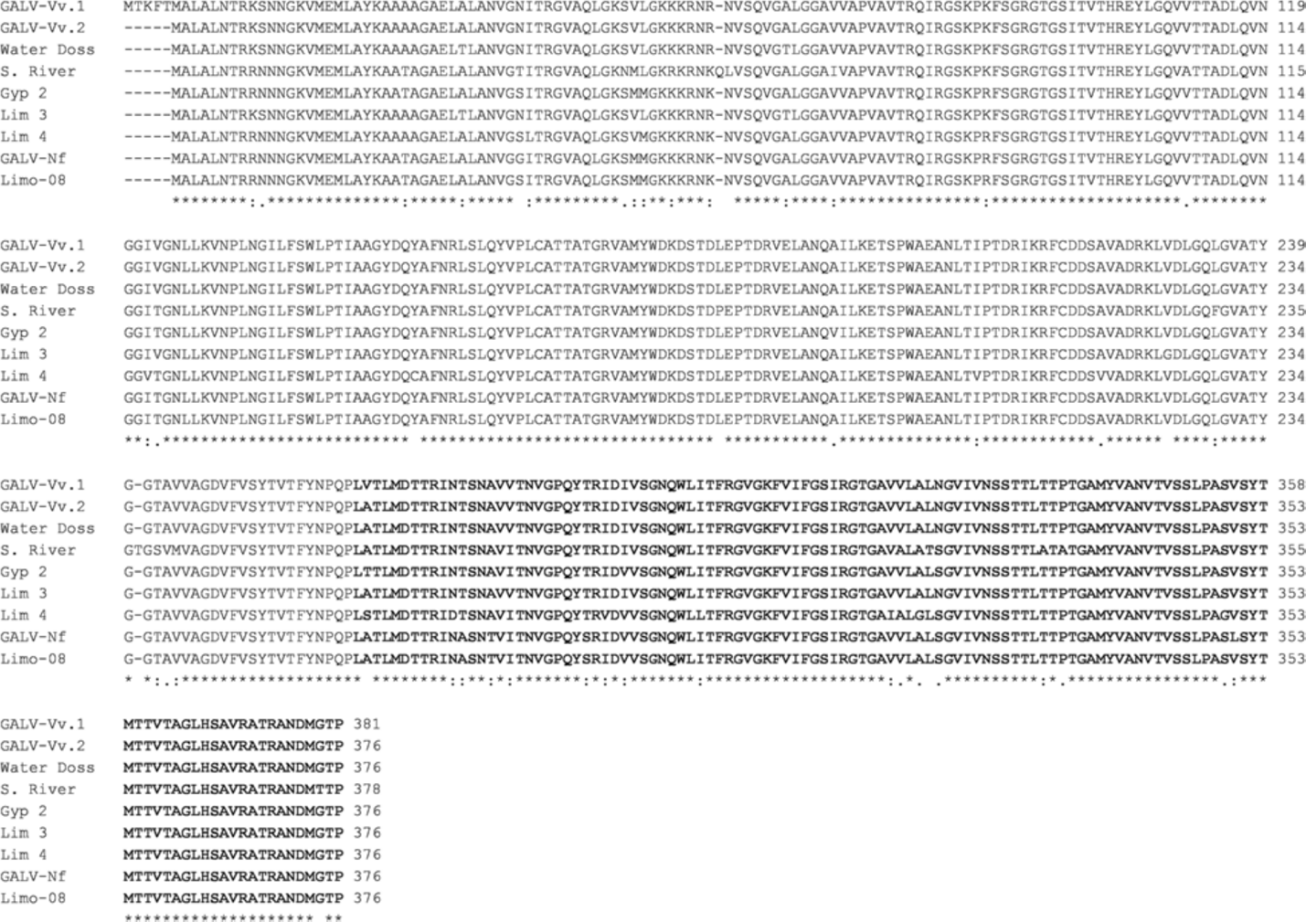
**Amino acid sequence alignment of predicted coat proteins derived from different GALV isolates.** Predicted protein sequences of different isolates (GALV-Vv.1, GALV-Vv.2, Water Doss, Schunter River (S. River), Gyp 2, Lim 3, Lim 4, GALV-Nf, Limo-08) were aligned. The protruding coat protein domains are shown in bold.

**Figure 2:**
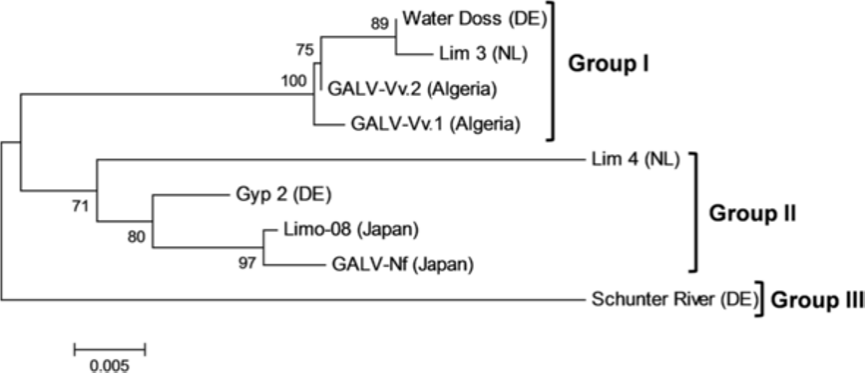
**Phylogenetic tree of predicted coat proteins derived from different GALV isolates.** Predicted protein sequences of different isolates were aligned and the phylogenetic three was constructed using the neighbor-joining method (from Algeria: GALV-Vv.1, GALV-Vv.2; from Germany (DE): Water Doss, Schunter River, Gyp 2; from Netherlands (NL): Lim 3, Lim 4 and from Japan: GALV-Nf, Limo-08).

The nucleotide and deduced amino acid sequences of the CP genes were aligned using ClustalW2 and a Percent Identity Matrix was produced in order to evaluate sequence similarities. The CP sequences from different isolates showed amino acid sequence identities ranging from 91.49 to 99.73% whereas the nucleotide sequence identities ranged from 84.03 to 99.73% (Table 1). The CP sequence lengths were also variable, comprising 376 amino acids in most cases but increasing to 378 and 381 residues for the Shunter River and GALV-Vv.1 isolates, respectively (Figure 1). Based on these available CP amino acid sequences, a phylogenetic tree was constructed, showing the sequences clustered in three distinct groups with high bootstrap values (Figure 2). The Water Doss, Lim 3 and GALV-Vv isolates clustered in group I. The Lim 4, Gyp 2, Limo-08 and GALV-Nf isolates clustered in group II. Finally, the Shunter River isolate was the sole representative of group III.

**Table 1:** **Percent identity matrix of amino acid and nucleotide coat protein sequences from different GALV isolates**

Isolates	GALV-Vv.1	GALV-Vv.2	Water doss	S. River	Gyp 2	Lim 3	Lim 4	GALV-Nf	Limo-08
**GALV-Vv.1**	**―**	99.73	99.20	93.35	96.28	99.94	93.88	95.48	95.74
**GALV-Vv.2**	99.91	**―**	99.47	93.62	96.28	98.2	93.88	95.74	96.01
**Water Doss**	99.13	**99.73**	**―**	93.09	95.74	**99.73**	93.35	95.21	95.48
**S. River**	85.08	85.5	85.29	**―**	94.41	92.82	**91.49**	93.88	94.15
**Gyp 2**	91.71	92.57	92.1	85.75	**―**	92.28	95.31	98.14	98.67
**Lim 3**	99.73	**99.73**	99.66	85.37	92.18	**―**	93.09	94.95	95.21
**Lim 4**	90.66	90.98	90.42	**84.03**	95.31	90.58	**―**	94.68	95.21
**GALV-Nf**	92.04	92.13	92.04	85.85	96.46	91.87	93.81	**―**	99.47
**Limo-08**	91.87	91.95	91.87	85.85	96.20	91.69	94.08	98.14	**―**

Deduced amino acid (above horizontal bars) and nucleotide (below horizontal bars) sequences from different isolates (from Algeria: GALV-Vv.1, GALV-Vv.2; from Germany: Water Doss, Schunter River (S. River), Gyp 2; from Netherlands: Lim 3, Lim 4 and from Japan: GALV-Nf, Limo-08) were aligned and a percent identity matrix was created. The lowest and the highest sequence identities between different isolates are shown in bold.

For completeness, the genomic sequences of the GALV-Vv [GenBank: KJ534082.1] and GALV-Nf [GenBank: AY830918.1] isolates were compared, revealing an overall identity of 93.46%.

### GALV epidemiological survey in a collection of grapevine samples

We analyzed 152 cDNAs from grapevine accessions of diverse provenance for the presence of GALV, including grapevine cultivars from commercial vineyards in the northern, central and southern regions of Italy and from germplasm collections of different grapevine genotypes from other countries such as Algeria, Germany, Portugal, Greece, Cyprus, Armenia, Slovakia, Romania and France (Additional file 1: Table S1). These samples comprised different American and Asiatic species commercially cultivated or used for breeding or rootstock production as well as a number of *V. vinifera* cultivars and accessions.

We screened the samples for the presence of GALV by RT-PCR, using a primer set designed to amplify a conserved portion of the GALV-Nf and GALV-Vv CP coding regions as well as the same CP fragment of most of the remaining GALV isolates, except Shunter River and Lim 4. No GALV infection was detected in any of the samples.

### Construction of full-length GALV-Nf cDNA clones

**Figure 3:**
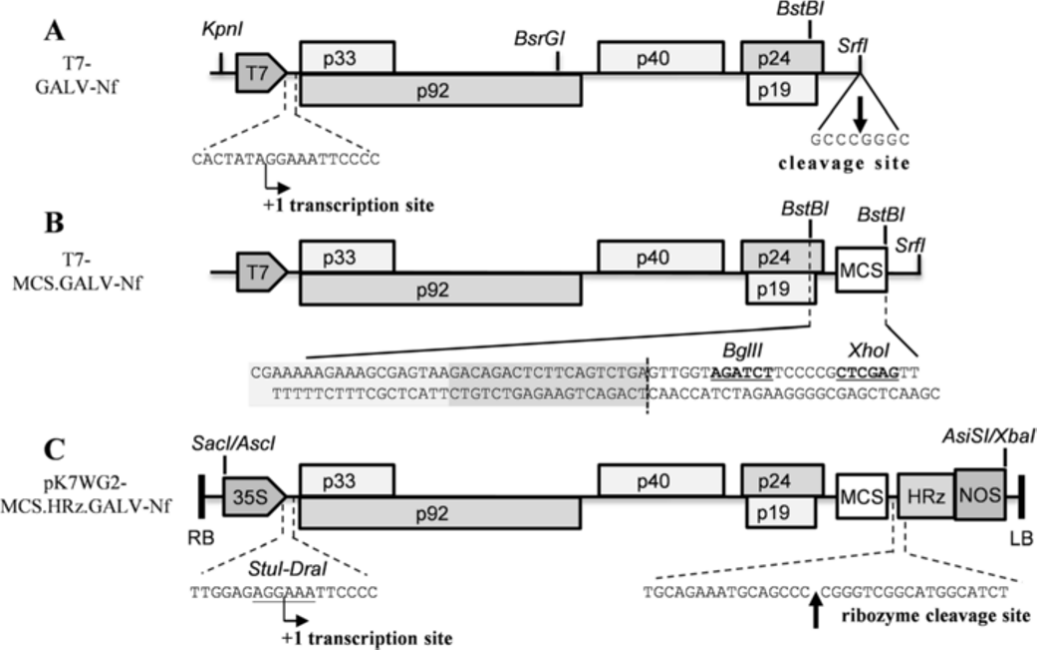
**Schematic representation of the infectious GALV-Nf cDNA clones.** GALV-Nf sequences under the control of T7 promoter (T7-GALV-Nf and T7-MCS.GALV-Nf) were linearized with SrfI and used for the *in vitro* production of infectious transcripts (details of T7 transcription site and SrfI cleavage site are shown in **panel A**). A polylinker was inserted by BstBI cleavage in the T7-GALV-Nf construct obtaining the T7-MCS.GALV-Nf vector (the BglII and XhoI sites of the polylinker are shown in bold and underlined in the sequence reported in **panel B**). The viral sequence, placed under the control of the CaMV 35S promoter (35S) and the *nos* terminator (NOS), was introduced (using SacI/AscI and AsiSI/XbaI sites) between the pK7WG2 left and right borders (RB and LB) producing the pK7WG2-MCS.HRz.GALV-Nf binary vector for *A. tumefaciens*-mediated infection. To design a functional 5′ viral end, the junction between the 35S and the viral sequence was obtained by ligating blunt-end fragments produced by digesting the 35S sequence with StuI and the viral sequence with DraI (details shown in **panel C**). The sequence of the *Hepatitis delta virus* antigenomic ribozyme (HRz) was introduced to allow the production of a correct 3′ viral end following ribozyme autocleavage **(panel C)**. T7: T7 promoter; p33 and p92: RNA-dependent RNA polymerases; p40: coat protein (CP); p24: movement protein (MP); p19: silencing suppressor.

We developed and tested three different infectious GALV clones (Figure 3A, B, C). First, two GALV-Nf partial sequences were synthesized and manipulated to produce the final T7-GALV-Nf vector (Figure 3A). This clone contains the full-length GALV-Nf sequence under the transcriptional control of the T7 promoter plus a SrfI site for vector linearization, which is required to produce the correct 3′ viral end by *in vitro* transcription [16, 17]. We added a multiple cloning site (MCS) downstream of the p24 coding region at the unique BstBI restriction site to create vector T7-MCS-GALV-Nf, which contains two new unique restriction sites (BglII and XhoI) thus allowing the further functionalization of this vector in future studies. Three different stop codons, one for each reading frame, were designed upstream the MCS to prevent the formation of aberrant viral proteins (Figure 3B). Finally, the pK7WG2-MCS.HRz.GALV-Nf binary vector was produced (using the SacI/AscI and AsiSI/XbaI restriction sites) placing the GALV-Nf sequence under the control of the CaMV 35S promoter and inserting the HRz ribozyme and *nos* terminator sequences immediately downstream of the viral 3′ UTR (Figure 3C). This vector can be delivered to plants by agroinfiltration and allows the production of GALV-Nf RNAs almost equivalent to the viral genome. In particular, by using the StuI-DraI ligation strategy described under Materials and Methods, a functional viral 5′ UTR sequence corresponding to the +1 transcriptional start site can be produced, lacking only two nucleotides from the original GALV-Nf genome sequence. The HRz ribozyme can produce the precise viral 3′ terminus by self-cleavage [11].

### Infectivity of GALV-Nf clones in *N. benthamiana*plants

**Figure 4:**
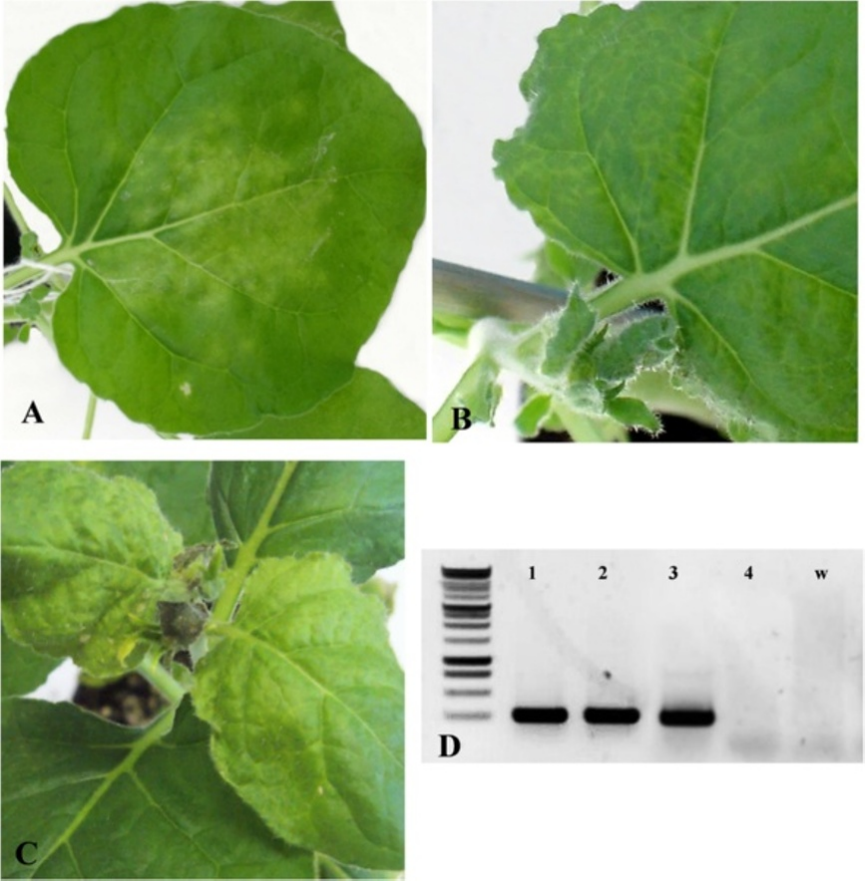
**Local and systemic GALV-Nf symptoms on** ***N. benthamiana*** **plants and GALV detection by RT-PCR. (A)** Local and **(B)** systemic GALV-Nf symptoms 7 days after the inoculation with T7-GALV-Nf viral transcripts and **(C)** systemic symptoms observed on *N. benthamiana* plants 12 days after agroinfiltration with the binary vector. **(D)** RT-PCR detection of GALV infection on systemic leaves of agroinfiltrated plants (**D** 1, 2, 3) and a healthy plant (**D** 4). w: water negative control.

The infectivity of GALV-Nf transcripts derived from the T7-GALV-Nf construct was investigated by rub-inoculating approximately 20 *N. benthamiana* plants with different lots of infectious transcripts. Coalescing chlorotic spots appeared on the inoculated leaves 4 days post-inoculation (dpi) (Figure 4A) followed by the development of systemic veinlet chlorosis at 7 dpi, starting from the proximal part of the leaf (Figure 4B). GALV-Nf infection was confirmed in systemically infected leaves by DAS-ELISA (data not shown). However, only ~30% of the plants were infected in each experiment.

**Figure 5:**
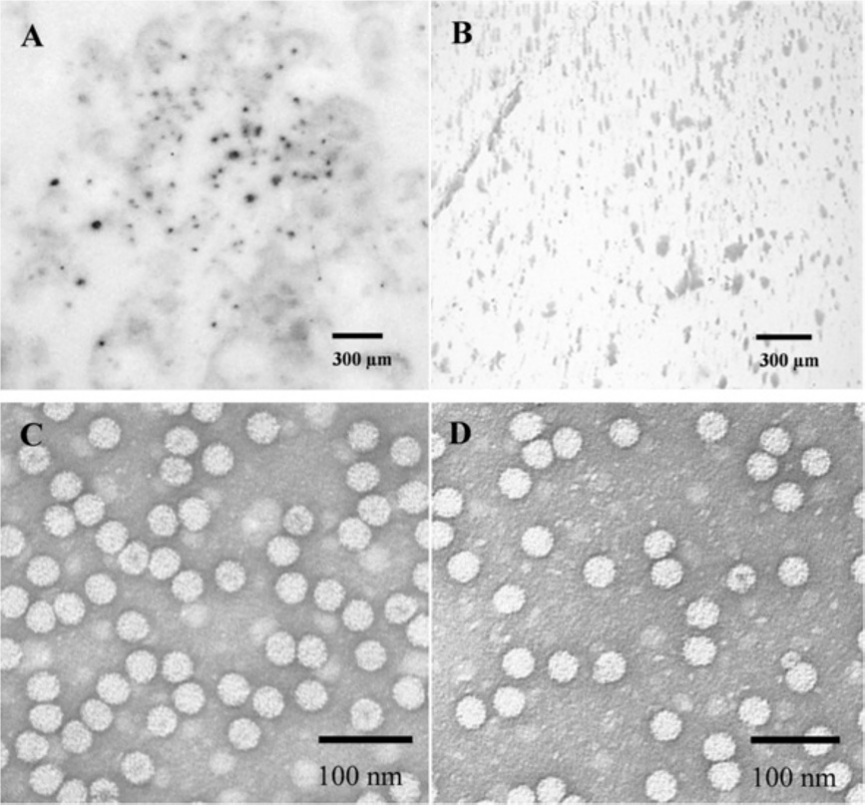
**Serological GALV-Nf detection in systemically** ***-*** **infected** ***N. benthamiana*** **leaves and analysis of viral particles by electron microscopy.** Tissue-print immunoassay using a GALV-specific antibody on systemically-infected leaves of **(A)** agroinfiltrated and **(B)** healthy plants. Purified viral particles from **(C)** T7-GALV-Nf infected or **(D)** agroinfiltrated *N. benthamiana* plants detected by immunosorbent electron microscopy.

We developed the GALV-Nf binary vector described above to improve the efficiency of infection, and delivered the vector to *N. benthamiana* plants by leaf agroinfiltration. Upper non-infiltrated leaves displayed light mottling with some necrotic spots, and apical leaf necrosis at 12 dpi (Figure 4C). The efficiency of infection in agroinfiltrated plants was ~90% in almost all experiments. Systemic spreading was confirmed in all symptomatic plants by RT-PCR analysis (Figure 4D) and by tissue-print immunoassays in the same plants (Figure 5A, B). GALV-Nf particles were purified from systemically infected leaves of *N. benthamiana* plants rubbed with the T7-GALV-Nf transcripts (Figure 5C) or following agroinfiltration with the binary vector (Figure 5D). Immunosorbent electron microscopy (ISEM) revealed in both cases the presence of correctly-assembled 32-nm particles with the expected icosahedral morphology.

### Cytopathology of *Agrobacterium*-mediated GALV-Nf infection in *N. benthamiana*plants

**Figure 6:**
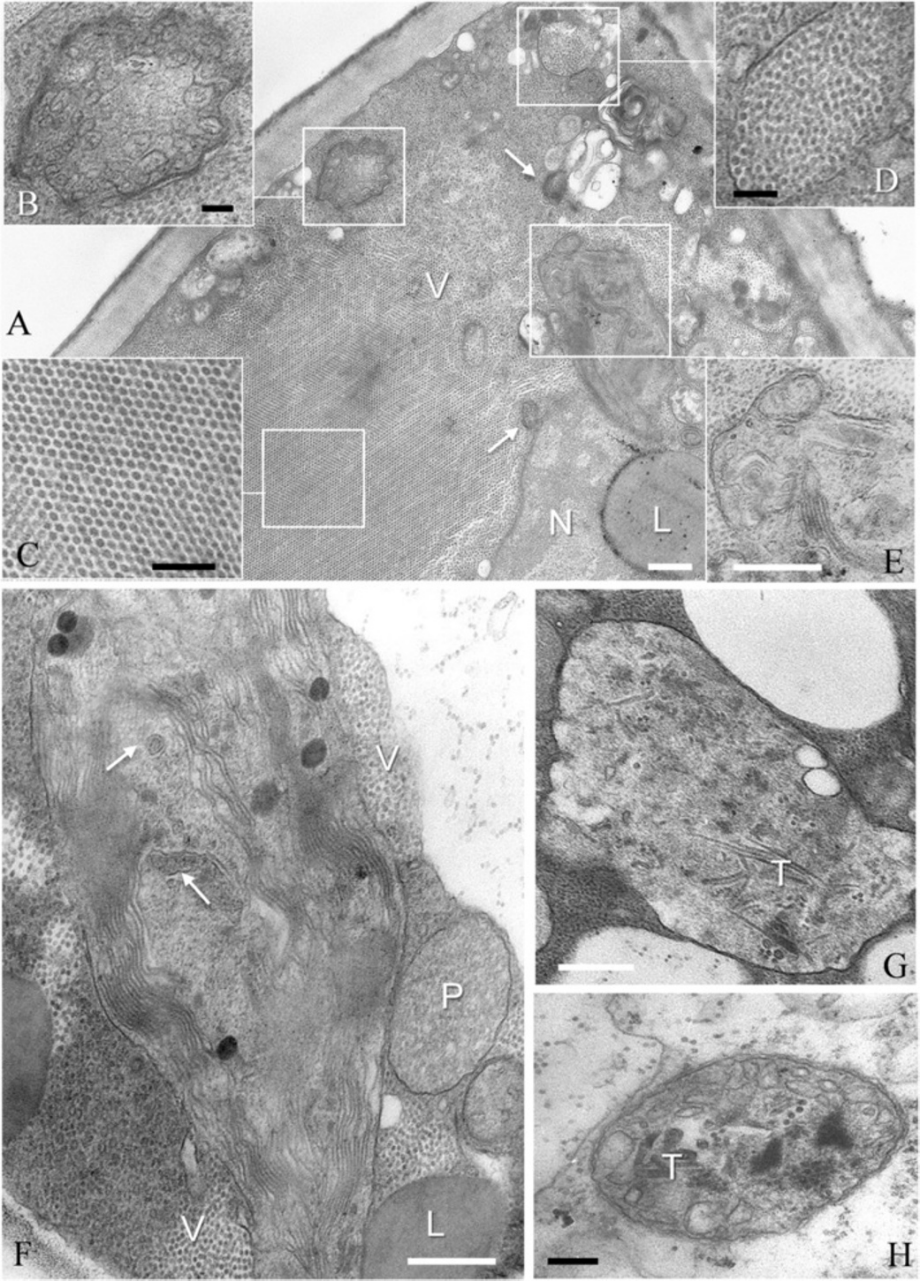
**GALV-Nf cytopathology in systemically-infected** ***N. benthamiana*** **leaves. (A)** Epidermal cell filled with virus particles (V) and showing some multivesicular bodies (arrows), one of them enlarged in **(B)**. Virions are distributed throughout the cytoplasm, or aggregated in a large crystal **(C)**, or enclosed in membrane-bound enclaves **(D)**. A plastid, enlarged in **(E)**, shows thylakoid disorganization and vesiculation. Similar chloroplast alterations are visible also in parenchyma mesophyll cells **(F)** and, in some instances, vesicles similar to those forming the multivesicular bodies are observed (arrows). In cells at a late stage of infection, the chloroplast structure appears completely disrupted **(G)** and electron-dense tubular structures (T) are visible in the stroma. These structures are sometimes present also in mitochondria **(H)**. N, nucleus; P, peroxisome; L, lipid globule. Black bars = 200 nm; white bars = 400 nm.

In systemically-infected leaves, icosahedral virus particles were present in almost all tissues, in the cytoplasm and vacuoles but not in other organelles (Figure 6). In some heavily-infected cells, large virus crystals occupied most of the cell lumen (Figure 6A, C) together with aggregates of virions in membrane-bound enclaves (Figure 6D). The other major cytopathic features of infected cells included the presence of multivesicular bodies formed by stacked vesicles containing a fibrillar network (Figure 6B) and altered chloroplasts showing thylakoid disorganization and vesiculation (Figure 6E). Thylakoid disorganization was observed in almost all chloroplasts of the parenchymal mesophyll cells. In some of these chloroplasts, vesicles similar to those forming the multivesicular bodies were also visible, either singly or in small groups, and stacked on the thylakoid membranes (Figure 6F). The analysis of multiple sections through the multivesicular bodies did not reveal the origin of these structures. In cells from leaves at the late stage of infection, the chloroplast structure was completely disrupted (Figure 6G) and electron-dense tubular structures were visible in the stroma. These structures were also present in some mitochondria (Figure 6H), although the morphology of the peroxisomes and mitochondria did not show significant changes in most of the infected cells.

### Infectivity of GALV-Nf in grapevine plants

**Figure 7:**
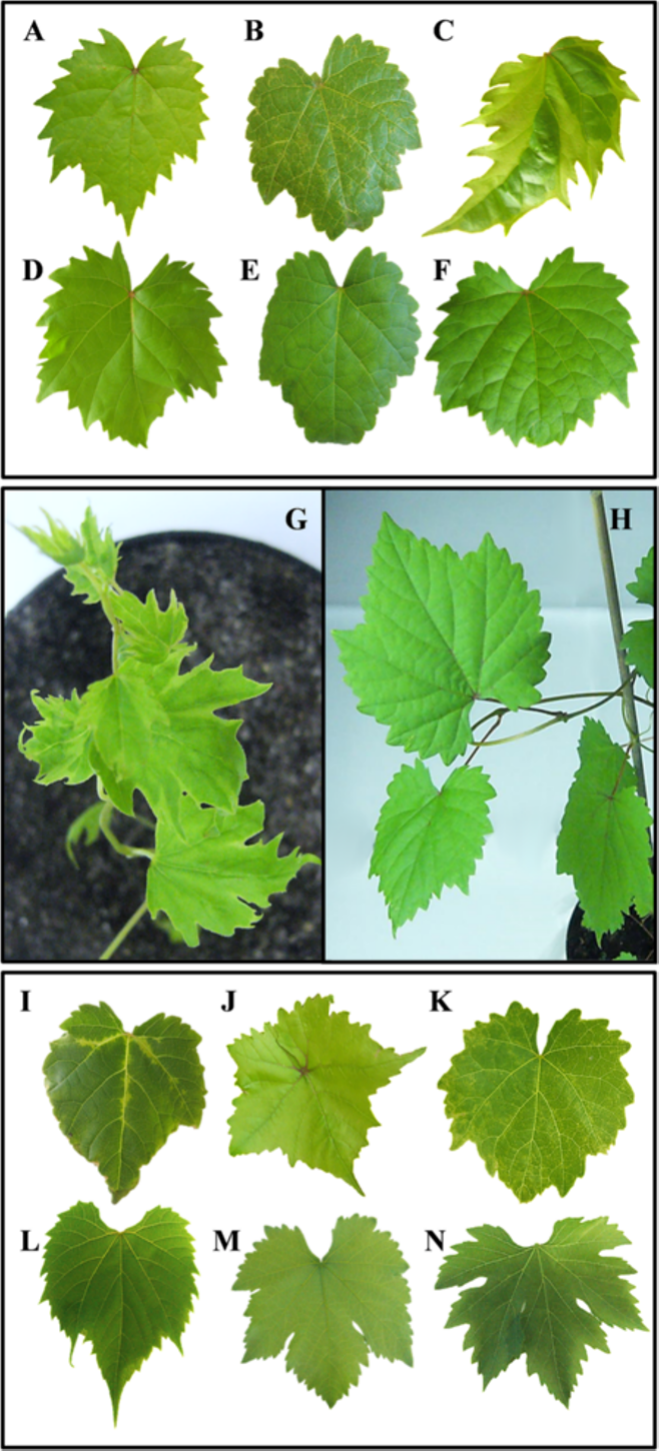
**Systemic GALV-Nf symptoms in grapevine plants.** GALV-Nf symptoms induced 5 weeks after infiltration with the GALV-Nf-based binary vector in different cultivars regenerated from somatic embryos: *V. vinifera* **(A)** Brachetto, **(B)** Syrah, **(C)** Nebbiolo compared to corresponding healthy plants **(D, E, F)**. Severe stunting and leaf alterations observed on a Nebbiolo plant **(G)** 5 weeks after infiltration with the GALV-Nf-based binary vector compared to a corresponding healthy plant **(H)**. GALV-Nf symptoms induced 5 weeks after infiltration with the GALV-Nf-based binary vector on different grapevine genotypes: **(I)** *V. riparia* cv. Gloire de Montpellier; *V. vinifera* **(J)** Sultana, **(K)** Corvina, compared to corresponding GALV-free plants **(L, M, N)**.

Plantlets representing different grapevine genotypes were inoculated with the binary vector by agroinfiltration. It is important to assess symptom development in completely virus-free plants, so we investigated the outcomes of infection in virus-free plants regenerated from somatic embryos representing the cultivars Brachetto, Syrah and Nebbiolo, to provide solid evidence that GALV-Nf can produce symptoms following artificial infection. Different symptoms were observed in emerging leaves 5 weeks after infiltration (Figure 7A, B, C) and these symptoms were absent in the corresponding healthy plants (Figure 7D, E, F). Regenerated Brachetto plants occasionally developed small chlorotic or necrotic spots along the veins (Figure 7A) whereas Syrah plants showed mild vein clearing or mottling (Figure 7B). No other developmental abnormalities were observed in either cultivar. In contrast, Nebbiolo plants showed malformations of the whole leaf lamina, chlorotic patches and dark green blistering on the leaves (Figure 7C) together with weak and stunted shoot growth (Figure 7G). These symptoms were not observed in healthy controls (Figure 7H).

Additional grapevine genotypes were tested for symptom development, i.e. *V. vinifera* cv. Sultana and cv. Corvina derived from certified mother-plants, and *V. riparia* cv. Gloire de Montpellier. In each case, plant growth was unaffected, shoots developed normally and the plants reached the same size and growing habits as uninfected controls. Mild systemic symptoms were visible only on emerging leaves about 5 weeks after infiltration (Figure 7I, J, K), and these were absent in GALV-free plants of the same genotypes (Figure 7L, M, N). We observed irregular chlorotic vein banding of the major leaf veins in *V. riparia* plants (Figure 7I), a slight upward curling of leaf margins in Sultana plants (Figure 7J) and light mottling in Corvina leaves (Figure 7K). These symptoms were consistent across all experiments, with an infection efficiency of ~90%, suggesting that artificial GALV-Nf infection can lead to the development of different symptoms in different genotypes.

**Figure 8:**
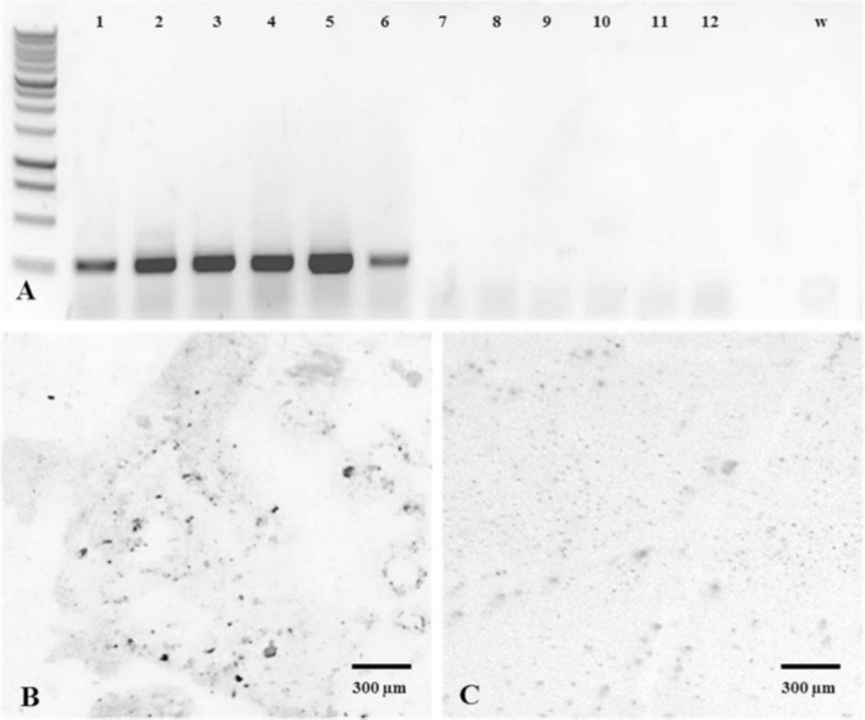
**Detection of GALV-Nf in systemically-infected grapevine plants. (A)** RT-PCR analysis of pK7WG2-MCS.HRz.GALV-Nf infected systemic leaves of (1) *V. riparia*, or *V. vinifera* cv. (2) Sultana, (3) Corvina, (4) Nebbiolo, (5) Syrah, (6) Brachetto, and on corresponding non-inoculated plants (7, 8, 9, 10, 11, 12). w: water negative control. Tissue-print immunoassay on systemically-infected Corvina leaf **(B)** and on a GALV-free leaf **(C)**.

The systemic infection of grapevine plants by GALV-Nf was confirmed by RT-PCR analysis of apical leaves representing the six infected genotypes (Figure 8A) and by tissue-print immunoassay (Figure 8B, C) confirming that the virus can replicate and spread systemically in all the cultivars we tested.

### Cytopathology of *Agrobacterium*-mediated GALV-Nf infection in grapevine

**Figure 9:**
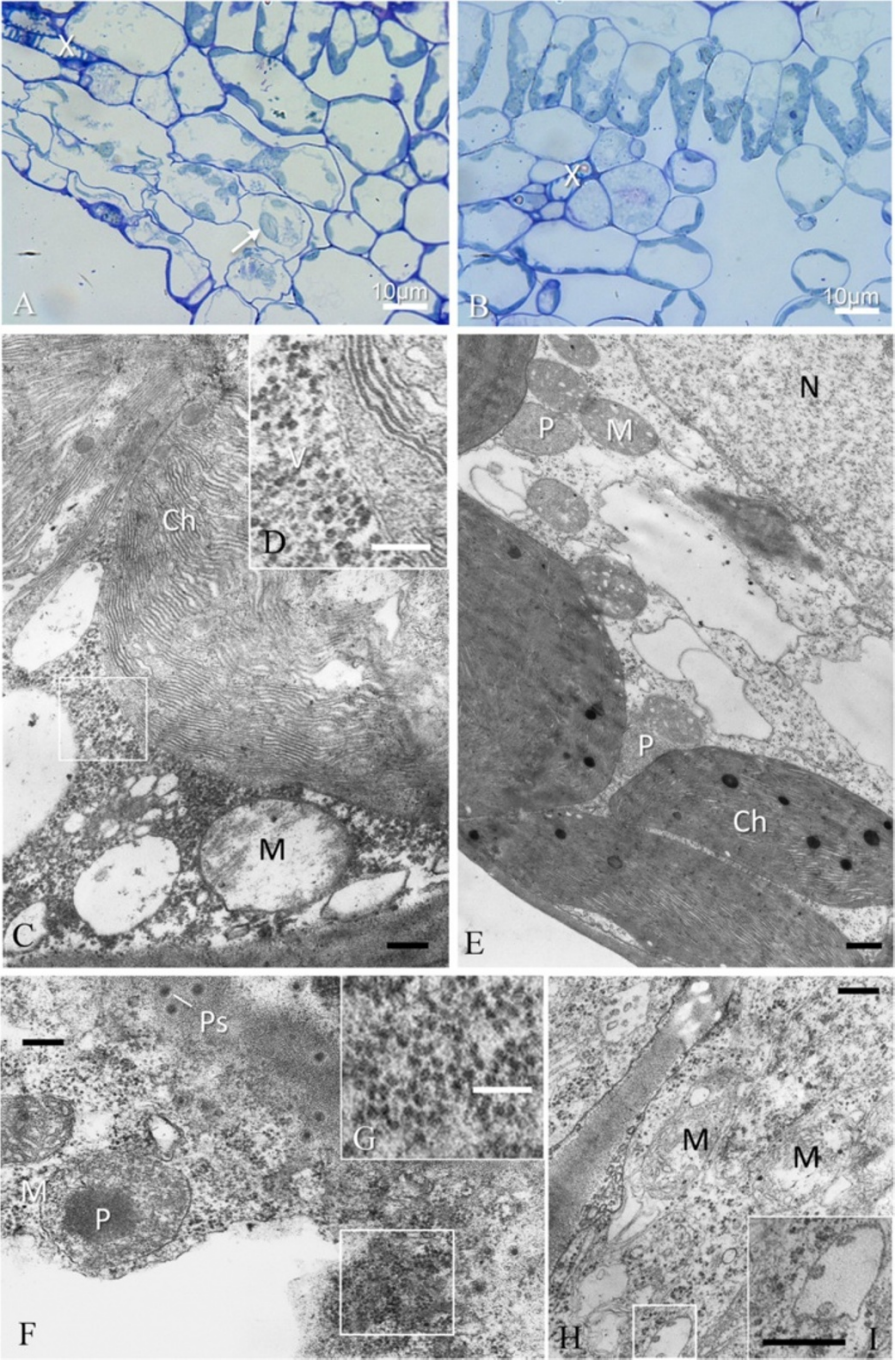
**GALV-Nf cytopathology of systemically-infected grapevine leaves. (A)** Virus infected cells are easily identified even under a light microscope close to small veins (X) and show different stages of plasmolysis and membrane rupture. Chloroplasts of spongy mesophyll (arrow) appear swollen, in comparison with those visible in an uninfected control leaf **(B)**. At the ultrastructural level **(C)**, swollen chloroplasts (Ch) show thylakoid disorganization, and numerous virus particles (V) are visible in the surrounding cytoplasm **(D)**; mitochondria (M) are also swollen, apparently without stroma and almost devoid of cristae. An uninfected control cell is visible in **(E)** for comparison. Cells around small veins sometimes show incipient necrosis **(F)** with dense cytoplasm in which virus-like particles are still distinguishable **(G)**. Infected parenchyma cells of small veins **(H)** show mitochondria with vacuolization and loss of cristae and small vesicles are sometimes present in apparently dilated ER cisternae **(I)**. N, nucleus; P, peroxisome; Ps, plasmodesmata. Black bars = 400 nm; white bars = 100 nm, if not otherwise stated.

Ultrastructural analysis of systemically-infected leaves (mainly in the Syrah plants showing vein clearing and mottling) confirmed that the most severely affected cells were those adjacent to small veins, particularly in the spongy mesophyll (Figure 9A). These cells showed different stages of plasmolysis and membrane rupture, and the chloroplasts appeared swollen compared to matched, uninfected control leaves (Figure 9B, E). At the ultrastructural level, the altered chloroplasts showed evidence of thylakoid disorganization, although without vesiculation (Figure 9C), and numerous virus particles were spread throughout the surrounding cytoplasm (Figure 9D). The mitochondria were occasionally swollen, and almost devoid of cristae and stroma (Figure 9C). Cells around small veins sometimes showed incipient necrosis (Figure 9F), and contained dense cytoplasm in which it was still possible to distinguish virus-like particles (Figure 9G). The mitochondria of infected parenchyma cells in small veins were vacuolated and devoid of cristae (Figure 9H) and small vesicles were sometimes present in dilated cisternae derived from the endoplasmic reticulum (Figure 9I). However, multivesicular bodies like those observed in *N. benthamiana* were not observed in the infected grapevine cells. All the ultrastructural alterations described above were also present in the symptomatic leaves of Nebbiolo plants. The only ultrastructural feature associated with the peculiar symptoms observed in Nebbiolo plants was the significant reduction in chloroplast number in cells representing the light-green areas of the leaf (data not shown).

## Discussion

### GALV is detected occasionally in the environment

GALV is a tombusvirus that can cause severe symptoms in certain plants, including nipplefruit and statice [6, 7], but it has been isolated only sporadically over the two decades since the discovery of the original grapevine isolate in 1986 [1]. It is most often found in environmental waters, a feature common to other water-borne plant viruses that can persist outside the host for a long time [18]. Natural GALV infections occur with a low incidence in most countries and the virus is not considered a threatening pathogen by the European and Mediterranean Plant Protection Organization (EPPO). Accordingly, our extensive survey of 152 grapevine plants from different locations showed no evidence of naturally-occurring GALV infection. GALV was also not detected in deep sequencing experiments carried out on leaves, petioles and phloem scrapes collected from grapevines showing symptoms of viral disease [19–22].

### GALV coat protein sequences have diversified into three major clusters

The paucity of natural GALV samples means that few sequences have been deposited in public databases. When our experiments began there were only seven GALV coat protein sequences from different isolates in the NCBI Nucleotide Database [5–7, 23], all identified based on biological features and serological reactions against an antibody produced against the original 1986 isolate [1]. There was only one full-length GALV genome sequence from a nipplefruit isolate [6] although the genome sequence of the original 1986 GALV isolate [1] was deposited during the course of our work [GenBank: KJ534082.1]. The sequences were highly conserved across isolates as anticipated, but as previously reported there were minor differences in the length of the CP open reading frame [5]. These minor differences do not prevent the original antiserum cross-reacting with all known isolates [1–6]. We found that the CP sequences clustered into three major groups but these did not correlate with the geographic origin, with the exception of the Japanese isolates which tended to show particularly strong conservation.

### A functional GALV genome can be created by synthetic biology

Viruses have been used to demonstrate the potential of synthetic biology because they have small genomes that can be synthesized *de novo* with great precision. The principle was demonstrated by synthesizing an artificial replicon based on *Hepatitis C virus* [24] and this was followed by the complete fabrication of poliovirus [25] and bacteriophage ϕX174 [26]. Plant viruses are particularly suitable for this approach because the majority possess small ssRNA(+) genomes that can be manipulated as cDNA and transcribed *in vitro* or *in planta*, and synthetic biology is therefore the ideal solution if no natural template is available, as is the case for GALV. Thus far, only *Tobacco mosaic virus* (TMV) has been synthesized *de novo*, although the initial synthetic genome was not infectious due to the presence of errors in the original sequence deposited in 1982 [27]. The authors aligned the original sequence with more recent strains and identified the changes required to synthesize an infectious clone [28]. Chimeric sequences were also tested to determine the regions of the viral genome responsible for pathogenicity and host range [28]. Improvements in DNA sequencing and synthesis now make such errors increasingly unlikely and reduce the need for the detailed genetic analysis of synthetic genomes [28]. We therefore used synthetic biology to produce infectious clones based on the GALV-Nf genome sequence and tested their ability to infect *N. benthamiana* and grapevine plants.

### The agroinfiltration of *N. benthamiana*leaves with infectious cDNA clones is more efficient than rub-inoculation with infectious transcripts

Initially we developed an infectious GALV-Nf cDNA clone for *in vitro* transcription, including a unique SrfI restriction site to define the 3′ end of the genome when the clone was linearized and thus generate the correct terminus. The construct also preserved the endogenous viral *cis*-acting elements that control replication. However, rub-inoculation with infectious transcripts only achieved an infection efficiency of ~30%, probably due to RNA degradation or a low frequency of complete transcription. We therefore developed a binary vector based on the same sequence, which was delivered to *N. benthamiana* plants by agroinfiltration and achieved an infection efficiency of ~90%. The improvement may have reflected a combination of the delivery method and the introduction of the HRz ribozyme sequence upstream the *nos* terminator, which has previously been shown to improve the infection efficiency of a TBSV vector and avoid the creation of a poly(A) tail *in planta* [11, 29]. The agroinoculation with GALV-Nf caused systemic infections with symptoms similar to those produced by the nipplefruit [6] and statice isolates [7] in *N. benthamiana*. Electron microscopy confirmed the assembly of viral particles identical to those derived from natural infections [1, 2, 6, 7, 30].

### The inoculation of grapevine plants with the GALV-Nf cDNA clone induces systemic infection

The inoculation of grapevine plants with GALV has never been reported [1, 6]. Having established that the GALV-Nf cDNA clone induces systemic infection in *N. benthamiana* plants, we tested the same construct on a variety of grapevine plants representing different species and cultivars, some of which were derived by somatic embryogenesis to avoid any potential interference from undetected viruses or viroids, thus ensuring that any symptoms are caused by GALV-Nf [31–33]. Where plants regenerated from somatic embryos were not available, we used *V. vinifera* rooted cuttings from certified mother plants that have been individually tested and indexed for the absence of all grapevine viruses indicated by current Italian legislation. GALV-Nf infection and spreading was demonstrated in all of the species and genotypes although the symptoms discussed below should be regarded as indicative and in need of further validation.

The different grapevine cultivars developed slightly different symptoms, which is consistent with previous reports showing that the severity of disease symptoms depends on plant species/cultivar, plant age, virus strain and the environment [34]. The infections produced mild symptoms in most cultivars, but Nebbiolo (derived by somatic embryogenesis) was exceptional, and the severity and peculiarity of the symptoms in these plants may make them useful as indicators for GALV infection [35]. We found that the GALV-Nf clone was able to infect, replicate and spread in all the different grapevine genotypes we tested and that the spreading was not limited to specific leaf tissues, as observed in other host plants [30].

### GALV-Nf causes specific cytopathological alterations in *N. benthamiana*and grapevine plants

The impact of GALV-Nf infection was investigated in more detail at the ultrastructural level by analyzing the symptomatic apical leaves of *N. benthamiana* and grapevine plants by electron microscopy. The ultrastructural alterations observed in infected *N. benthamiana* plants were similar to those induced by tombusviruses in other host plants [36], including the formation of large membrane-bound clusters of virions occupying most of the cell lumen (often in the form of crystals), the extensive vesiculation of chloroplasts and the disorganization of thylakoids. Rod-like structures were present in the stroma of chloroplasts and mitochondria, as previously observed in *C. quinoa* cells infected with GALV [3]. We also observed the formation of multivesicular bodies typical of tombusvirus infections [37, 38] but we were unable to determine the origin of these structures.

Similar cytopathological features were observed in grapevine but the ultrastructural changes were less dramatic, with evidence of virion clusters and swollen, disorganized chloroplasts, but no evidence of vesiculation or the formation of multivesicular bodies.

Multivesicular bodies derived from peroxisomes have been observed in plant cells infected with most tombusviruses, except for *Carnation Italian ringspot virus* (CIRV) and *Pelargonium necrotic spot virus* (PelNSV) infections, where the multivesicular bodies originated from the mitochondria [39]. The peripheral vesicles of the multivesicular bodies are thought to be sites of tombusvirus replication [9, 40, 41]. In *Tomato bushy stunt virus*, *Cymbidium ringspot virus* and *Cucumber necrosis virus*, peroxisomal targeting sequences have been identified in the N-terminal region of the p33 replication protein, which is responsible for template RNA recruitment to the site of replication [42, 43]. Although the GALV-Nf and TBSV p33 sequence is highly conserved, we were unable to detect multivesicular bodies in our infected grapevine plants under the experimental conditions we used. However, the targeting of TBSV p33/p92 to the peroxisome membrane was not sufficient to induce multivesicular bodies when these proteins were expressed in transgenic tobacco plants [38]. Instead, their ectopic accumulation led to the vesiculation of the peroxisomal boundary membrane resulting in the formation of novel cytosolic vesicles that were not topologically equivalent to those formed during the biogenesis of classical peroxisomal multivesicular bodies [38]. The formation of multivesicular bodies may therefore depend on other features of the viral sequence, other viral proteins, and on their compatibility with specific host proteins [37, 39]. Recent investigations have highlighted the important contribution of host proteins that are recruited by the virus to allow the assembly of the viral replication complex, including the formation of virus-induced vesicles [44–46]. Our initial findings require further investigation to determine whether specific features of either the grapevine cells and/or of the infectious construct may be responsible for the unique cytopathology we observed.

## Conclusions

We have produced a synthetic GALV-Nf cDNA clone and a corresponding binary vector which we used to induce the first artificial agroinfection of *N. benthamiana* and grapevine plants with GALV. Systemic GALV infections were characterized in terms of symptom development and ultrastructural changes in *N. benthamiana* and different grapevine genotypes revealing unique features of the infection in specific genotypes. We have shown that synthetic biology can be used to investigate the infectivity of viruses in plants even in cases where a source of the virus is unavailable. We will now be able to develop applications for this synthetic construct including recombinant protein expression and virus-induced gene silencing.

## Methods

### GALV sequence analysis

Eight GALV coat protein (CP) sequences from different isolates were sourced from the NCBI Nucleotide Database (http://www.ncbi.nlm.nih.gov/nuccore). The isolates were nipplefruit (GALV-Nf [GenBank: AAX76897.1]) [6], grapevine (GALV-Vv.1 [GenBank: AF540885.1]) [23], rivers (Shunter River [GenBank: AY500888.1] and Water Doss [GenBank: AY500878.1]) [5], groundwater in a statice production glasshouse (Lim 4 [GenBank: AY500884.1]) [5], statice (Lim 3 [GenBank: AY500883.1] [5], Limo-08 [GenBank: AB461854.1] [7]) and *Gypsophila paniculata* (Gyp 2 [GenBank: AY500880.1]) [5]. In addition, the complete GALV genome sequence from a nipplefruit isolate (GALV-Nf) was retrieved and used to produce the synthetic infectious clone [GenBank: AY830918.1]. Most recently, a complete GALV genome sequence from a grapevine isolate was also sequenced and deposited in the database [GenBank: KJ534082.1], providing an additional CP sequence (GALV-Vv.2 [GenBank: AHZ12757.1]).

Alignments and Percent Identity Matrices were obtained by ClustalW2 analysis (http://www.ebi.ac.uk/Tools/msa/clustalw2/). A phylogenetic tree was constructed using MEGA v5.2 [47] by the neighbor-joining method and node values were estimated by bootstrap analysis using 2000 replications. ClustalW2 was also used for the comparison between the GALV-Nf and GALV-Vv.

### Epidemiological GALV survey in a collection of grapevine samples

Vines growing in Italian commercial vineyards and germplasm collections (including grapevines from other countries) were surveyed for GALV. We tested 152 different cDNA samples by amplifying a 324-bp fragment of the GALV CP sequence. Primers 5′-GGG GAT GTG TTT GTC AGT TAC-3′ and 5′-GCT TGC CGG TAA TGA TGA TA-3′ were designed to allow the amplification of both GALV-Vv [GenBank: AHZ12757.1, AF540885.1] and GALV-Nf [GenBank: AAX76897.1]. The PCR products were analyzed by gel electrophoresis.

### Construction of the full-length GALV-Nf cDNA clones

Two GALV fragments composing the GALV-Nf genome sequence [GenBank:AY830918.1] were synthesized *de novo* along with the T7 promoter (TAATACGACTCACTATAGG) and cloned in two pUC57 vectors by Genscript (Piscataway, USA). The first fragment, containing the T7 promoter and the 2192-bp 5′ genome fragment was released from the first vector by digestion with KpnI/BsrGI and inserted, using the same enzymes, into the second vector containing the terminal 2541-bp 3′ genome fragment and the SrfI linearization site. This yielded the T7-GALV-Nf vector (Figure 3A). A 26-bp polylinker was inserted downstream of the p24 coding region at the BstBI site for future vector functionalization (Figure 3B) to yield vector T7-MCS.GALV-Nf.

The intermediate vector was fitted with the *Cauliflower mosaic virus* (CaMV) 35S promoter sequence [GenBank: GQ463722.1, missing the last three nucleotides] upstream of the viral genome by blunt-end ligation following digestion with StuI and DraI, and the *Hepatitis delta virus* ribozyme (HRz [48]) and the nopaline synthase (*nos*) terminator [GenBank: AF485783.1] downstream the viral 3′ UTR (Figure 3C). The CaMV 35S promoter was amplified using primers 5′-TTA ATT AAG GCG CGC CCC ATG GAG TCA AAG ATT CAA A-3′ and 5′-AAA GGC CTC TCC AAA TGA AAT AG-3′ and the *nos* terminator was amplified using primers 5′-GGA TCC GAT CGT TCA AAC ATT TGG CAA T-3′ and 5′-GCC CGG GCG CGA TCG CGA TCT AGT AAC ATA GAT GAC AC-3′. The HRz sequence was synthetized as oligonucleotides and annealed. The products were cloned in pGEM-T Easy (Promega, Madison, USA) and sequenced for verification then released and assembled into the T7-MCS.GALV-Nf by ligation. The GALV-Nf expression cassette (CaMV 35S promoter, GALV-Nf sequence, ribozyme and *nos* terminator) was then transferred, by digestion with AscI and AsiSI, into a pK7WG2 vector [49] previously modified by replacing the CaMV 35S promoter, *att* recombination sites, chloramphenicol resistance gene, 35S terminator and a portion of the kanamycin-resistance gene with a polylinker sequence including SacI/AscI and AsiSI/XbaI sites. The final pK7WG2-MCS.HRz.GALV-Nf vector was introduced into *Agrobacterium tumefaciens* strain EHA105 by electroporation (Bio-Rad, Hercules, CA, USA).

### Plant material and growth conditions

*N. benthamiana* plants were grown at 21–30°C in a greenhouse with a 15-h photoperiod and 32–50% relative humidity. *V. riparia* cv. Gloire de Montpellier plants were kindly provided by Julius Kühn-Institut (JKI), Institute for Grapevine Breeding Geilweilerhof–Siebeldingen, Germany. Certified mother plants of *V. vinifera* cv. Sultana and cv. Corvina were provided by Rauscedo nursery, Rauscedo (PN), Italy. *V. vinifera* cv. Syrah plants were kindly provided by Mandy Walker, CSIRO, Australia. *V. vinifera* cv. Brachetto and cv. Nebbiolo plants were regenerated from somatic embryos [31]. All grapevine plants were micropropagated *in vitro* and grown in a growing chamber at 25°C with a 16-h photoperiod. Two weeks after agroinfiltration, the plants were transferred to a peat-sand mixture in pots, and grown at 21°C with a 16-h photoperiod.

### *In vitro*transcription and agroinoculation

Freshly prepared *in vitro* transcripts of GALV-Nf were obtained from 1 μg of T7-GALV-Nf linearized with SrfI and transcribed using the MEGAscript® T7 Transcription Kit (Ambion, Austin, TX). Leaves of 5-week-old *N. benthamiana* plants were mechanically inoculated by gently scrubbing each leaf with 5–6 μg of infectious transcripts mixed with a small amount of celite (400 mesh). Two leaves from at least three plants were inoculated in each experiment, and all experiments were carried out five times.

For leaf agroinfiltration, *A. tumefaciens* EHA105 cells carrying the pK7WG2-MCS.HRz.GALV-Nf vector were grown for 2 days on solid Luria-Bertani (LB) medium supplemented with 300 μg/ml streptomycin, 100 μg/ml spectinomycin and 50 μg/ml rifampicin. A single colony was inoculated into a 50-ml liquid LB medium containing the appropriate antibiotics and grown overnight at 28°C with constant shaking. Cells were harvested by centrifugation at 6000 × *g* for 10 min at room temperature, resuspended in infiltration medium (10 mM MES pH 5.8, 10 mM MgCl_2_ and 100 μM acetosyringone), adjusted to an OD_600_ of 0.4 and left at room temperature for 3 h. Two fully-expanded *N. benthamiana* leaves were infiltrated using a needleless 5-ml syringe. For grapevine agroinfiltration, *in vitro* plants grown in glass tubes were immersed in the bacterial suspension and vacuum infiltrated (2× 2 min, 90 kPa). After agroinfiltration, plantlets were rinsed with sterile water and allowed to recover *in vitro* for 2 weeks before acclimation to *ex vitro* conditions. We used 6–8 plants per experiment, with five replicates.

### Tissue-print western analysis and particle purification

Leaves from infected or uninfected (control) plants were placed onto a nitrocellulose membrane, overlain with contact paper and blotted by applying a rolling pin. The membrane was air dried and incubated for 30 min with 1% periodic acid to block endogenous peroxidases [50, 51]. Serological GALV-Nf detection on the tissue-print was carried out using anti-GALV polyclonal antibodies (DSMZ, Braunschweig, Germany) diluted 1:5,000 and bound primary antibody was detected with a peroxidase-conjugated goat anti-rabbit secondary antibody A6154 (Sigma-Aldrich Corporation, St. Louis, MO, USA) diluted 1:15,000. The signal was developed with DAB Peroxidase substrate (Sigma FAST™ 3,3′-diaminobenzidine tetrahydrochloride with metal enhancer, Sigma-Aldrich Corporation, St. Louis, MO, USA) and images were captured under a Leica MZ16F stereomicroscope equipped with a Leica DFC420 C camera. GALV-Nf particles were purified from systemically-infected *N. benthamiana* as previously described for TBSV [29].

### RNA extraction and RT-PCR

Total RNA was extracted from systemically infected *N. benthamiana* leaves 12 days after infiltration using TRIzol® Reagent (Invitrogen, Carlsbad, CA, USA), and from the apical leaves of grapevine plants 5 weeks after infiltration using the Spectrum™ Plant Total RNA Kit (Sigma-Aldrich Corporation, St. Louis, MO, USA) according to the manufacturers’ protocols. RNA was treated with TURBO DNA-free™ (Ambion, Austin, TX) to remove DNA contamination and first-strand cDNA was synthesized using 2 μg RNA, 200 ng random primers (Promega, Madison, USA) and SuperScript™ III Reverse Transcriptase (Invitrogen, Carlsbad, CA, USA). To confirm GALV-Nf infection, a 265-bp fragment of the CP coding region was amplified with GoTaq® DNA Polymerase (Promega, Madison, USA) using primers 5′-GGG ATG TGT TTG TCA GTT AC-3′ and 5′-GGT AAG GGT AGT GGA GGA G-3′ followed by agarose gel electrophoresis.

### Transmission electron microscopy of purified particles and GALV-Nf cytopathology

GALV-Nf particles purified from *N. benthamiana* plants were analyzed by immunosorbent electron microscopy (ISEM) as described [52]. Briefly, carbon and celloidin coated 400 mesh copper grids were pre-incubated for 30 minutes with the anti-GALV polyclonal antibody (DSMZ, Braunschweig, Germany) diluted 1:1,000 and then washed five times with PBS (pH 7). Coated grids were incubated over a drop of GALV-Nf suspension for several minutes, then washed with five drops of 2% uranyl acetate and observed in a JEOL 100SX Transmission Electron Microscope. Tissue fragments (1–2 mm^2^) for ultrastructural studies were cut from systemically-infected *N. benthamiana* and grapevine leaves and fixed in 1.2% glutaraldehyde and 3.3% paraformaldehyde in 0.1 M phosphate buffer pH 7.4 at 4°C for 2 h, post-fixed in 1% OsO_4_ in the same buffer for 2 h, dehydrated in an ethanol series and embedded in Spurr’s resin [53]. Sections were stained with toluidine blue for light microscopy and with uranyl acetate and lead citrate for TEM.

## Electronic supplementary material

Additional file 1: Table S1: Detection of GALV in different grapevine plants by RT-PCR. Survey of GALV in 152 grapevine samples from different geographic areas. (PDF 39 KB)

## References

[1] Gallitelli D, Martelli GP, Di Franco A (1989). *Grapevine Algerian latent virus*, a newly recognized *Tombusvirus*. Phytoparasitica.

[2] Cannizzaro G, Rosciglione B, Castellano MA (1990). The presence of phytopathogenic viruses in waterways of western Sicily. Inf Fitopatol.

[3] Yi L, Lesemann DE, Koenig R, Rüdel M, Pfeilstetter E (1992). Isometric plant viruses in ditches and streams in agricultural areas: recovery of previously found viruses and identification of hitherto unrecorded *Carmo*- and *Tombusviruses* including *Grapevine Algerian latent virus*. J Phytopathol.

[4] Fuchs E, Grüntzing M, Auerbach I, Einecke I, Müller C, Krägenow M (1994). On the occurrence of plant pathogenic viruses in waters in the region of Halle/Saale (German Federal State of Saxony-Anhalt). Arch Phytopathol Pflanzenschutz.

[5] Koenig R, Verhoeven JTJ, Fribourg CE, Pfeilstetter E, Lesemann DE (2004). Evaluation of various species demarcation criteria in attempts to classify ten new *Tombusvirus* isolates. Arch Virol.

[6] Ohki T, Uematsu S, Nakayama Y, Lesemann DE, Honda Y, Tsuda S, Fujisawa I (2006). Characterization of *Grapevine Algerian latent virus* isolated from nipplefruit (*Solanum mammosum*) in Japan. J Gen Plant Pathol.

[7] Fujinaga M, Ogiso H, Wakabayashi H, Morikawa T, Natsuaki T (2009). First report of a *Grapevine Algerian latent virus* disease on statice plants (*Limonium sinuatum*) in Japan. J Gen Plant Pathol.

[8] Kim S, Cho WK, Lee HG, Park SH, Sohn SH, Kim KH (2012). The p19 protein of *Grapevine Algerian latent virus* is a determinant of systemic infection of *Chenopodium quinoa*. Virus Res.

[9] White KA, Nagy PD (2004). Advances in the molecular biology of *Tombusviruses*: gene expression, genome replication, and recombination. Prog Nucleic Acid Res Mol Biol.

[10] Scholthof HB, Scholthof KB, Jackson AO (1996). Plant virus gene vectors for transient expression of foreign proteins in plants. Annu Rev Phytopathol.

[11] Scholthof HB (1999). Rapid delivery of foreign genes into plants by rub-inoculation with intact plasmid DNA of a *Tomato bushy stunt virus* gene vector. J Virol.

[12] Hou H, Qiu W (2003). A novel co-delivery system consisting of a *Tomato bushy stunt virus* and a defective interfering RNA for studying gene silencing. J Virol Methods.

[13] Pignatta D, Kumar P, Turina M, Dandekar A, Falk BW (2007). Quantitative analysis of efficient endogenous gene silencing in *Nicotiana benthamiana* plants using *Tomato bushy stunt virus* vectors that retain the capsid protein gene. Mol Plant Microbe Interact.

[14] Scholthof HB, Jackson AO (1997). The enigma of pX: a host-dependent *cis*-acting element with variable effects on *Tombusvirus* RNA accumulation. Virology.

[15] Scholthof HB (2006). The *Tombusvirus*-encoded P19: from irrelevance to elegance. Nat Rev Microbiol.

[16] Fabian MR, Na H, Ray D, White KA (2003). 3′-Terminal RNA secondary structures are important for accumulation of *Tomato bushy stunt virus* DI RNAs. Virology.

[17] Nagy PD, Pogany J (2000). Partial purification and characterization of *Cucumber necrosis virus* and *Tomato bushy stunt virus* RNA-dependent RNA polymerases: similarities and differences in template usage between *Tombusvirus* and *Carmovirus* RNA-dependent RNA polymerases. Virology.

[18] Mehle N, Ravnikar M (2012). Plant viruses in aqueous environment – survival, water mediated transmission and detection. Water Res.

[19] Giampetruzzi A, Roumi V, Roberto R, Malossini U, Yoshikawa N, La Notte P, Terlizzi F, Credi R, Saldarelli P (2012). A new grapevine virus discovered by deep sequencing of virus- and viroid- derived small RNAs in cv. Pinot Gris. Virus Res.

[20] Coetzee B, Freeborough MJ, Maree HJ, Celton JM, Rees DJG, Burger JT (2010). Deep sequencing analysis of viruses infecting grapevines: virome of a vineyard. Virology.

[21] Pantaleo V, Saldarelli P, Miozzi L, Giampetruzzi A, Gisel A, Moxon S, Dalmay T, Bisztray G, Burgyan J (2010). Deep sequencing analysis of viral short RNAs from an infected Pinot Noir grapevine. Virology.

[22] Al Rwahnih M, Daubert S, Golino D, Rowhani A (2009). Deep sequencing analysis of RNAs from a grapevine showing Syrah decline symptoms reveals a multiple virus infection that includes a novel virus. Virology.

[23] Russo M, Vovlas C, Rubino L, Grieco F, Martelli GP (2002). Molecular characterization of a *Tombusvirus* isolated from diseased pear trees in southern Italy. J Plant Pathol.

[24] Blight KJ, Kolykhalov AA, Rice CM (2000). Efficient initiation of HCV RNA replication in cell culture. Science.

[25] Cello J, Paul AV, Wimmer E (2002). Chemical synthesis of poliovirus cDNA: generation of infectious virus in the absence of natural template. Science.

[26] Smith HO, Hutchison CA, Pfannkoch C, Venter JC (2003). Generating a synthetic genome by whole genome assembly: ΦX174 bacteriophage from synthetic oligonucleotides. Proc Natl Acad Sci U S A.

[27] Cooper B (2014). Proof by synthesis of *Tobacco mosaic virus*. Genome Biol.

[28] Wimmer E, Paul AV (2011). Synthetic poliovirus and other designer viruses: what have we learned from them?. Annu Rev Microbiol.

[29] Grasso S, Lico C, Imperatori F, Santi L (2013). A plant derived multifunctional tool for nanobiotechnology based on *Tomato bushy stunt virus*. Transgenic Res.

[30] Brunt AA, Crabtree K, Dallwitz MJ, Gibbs AJ, Watson L (1996). Viruses of Plants. Descriptions and Lists from the VIDE Database.

[31] Gambino G, Bondaz J, Gribaudo I (2006). Detection and elimination of viruses in callus, somatic embryos and regenerated plantlets of grapevine. Eur J Plant Pathol.

[32] Wild J, Kasdorf G (1991). The effectiveness of *in vitro* somatic embryogenesis in eliminating fanleaf virus and leafroll associated viruses from grapevines. S Afr J Enol Vitic.

[33] Gambino G, Di Matteo D, Gribaudo I (2009). Elimination of *Grapevine fanleaf virus* from three *Vitis vinifera* cultivars by somatic embryogenesis. Eur J Plant Pathol.

[34] Walter B, Martelli G (1998). Consideration on grapevine selection and certification. Vitis.

[35] Martelli GP, Savino V, Walter B, Martelli GP (1993). Indexing on *Vitis* Indicators. Graft-Transmissible Diseases of Grapevines: Handbook for Detection and Diagnosis.

[36] Martelli GP, Gallitelli D, Russo M, Koenig R (1988). Tombusviruses. The Plant Viruses; Polyhedral virions with monopartite RNA genomes, Volume 2.

[37] Russo M, Di Franco A, Martelli GP (1987). Cytopathology in the identification and classification of *Tombusviruses*. Intervirology.

[38] McCartney AW, Greenwood JS, Fabian MR, White KA, Mullen RT (2005). Localization of the *Tomato bushy stunt virus* replication protein p33 reveals a peroxisome-to-endoplasmic reticulum sorting pathway. Plant Cell.

[39] Koenig R, Lesemann DE (2009). New isolates of *Carnation Italian ringspot virus* differ from the original one by having replication-associated proteins with a typical *Tombusvirus*-like N-terminus and by inducing peroxisome- rather than mitochondrion-derived multivesicular bodies. Arch Virol.

[40] Scholthof KB, Scholthof HB, Jackson A (1995). The *Tomato bushy stunt virus* replicase proteins are coordinately expressed and membrane associated. Virology.

[41] Rubino L, Russo M (1998). Membrane targeting sequences in *Tombusvirus* infections. Virology.

[42] Monkewich S, Lin HX, Fabian MR, Xu W, Na H, Ray D, Chernysheva OA, Nagy PD, White KA (2005). The p92 polymerase coding region contains an internal RNA element required at an early step in *Tombusvirus* genome replication. J Virol.

[43] Pogany J, White KA, Nagy PD (2005). Specific binding of *Tombusvirus* replication protein p33 to an internal replication element in the viral RNA is essential for replication. J Virol.

[44] Mullen RT, McCartney AW, Flynn CR, Smith GS (2006). Peroxisome biogenesis and the formation of multivesicular peroxisomes during *Tombusvirus* infection: a role for ESCRT?. Can J Bot.

[45] Pogany J, Stork J, Li Z, Nagy PD (2008). *In vitro* assembly of the *Tomato bushy stunt virus* replicase requires the host Heat shock protein 70. Proc Natl Acad Sci U S A.

[46] Nagy PD, Pogany J (2012). The dependence of viral RNA replication on co-opted host factors. Nat Rev Microbiol.

[47] Tamura K, Peterson D, Peterson N, Stecher G, Nei M, Kumar S (2011). MEGA5: molecular evolutionary genetics analysis using maximum likelihood, evolutionary distance, and maximum parsimony methods. Mol Biol Evol.

[48] Sharmen L, Kuo MYP, Dinter-Gottlieb G, Taylor J (1988). Antigenomic RNA of human *Hepatitis delta virus* can undergo self-cleavage. J Virol.

[49] Karimi M, Inzé D, Depicker A (2002). GATEWAY vectors for *Agrobacterium*-mediated plant transformation. Trends Plant Sci.

[50] Pereira S, Carvalho H, Sunkel C, Salema R (1992). Immunocytolocalization of glutamine synthetase in mesophyll and phloem of leaves of *Solanum tuberosum* L. Protoplasma.

[51] Carvalho H, Pereira S, Sunkel C, Salema R (1992). Detection of a cytosolic glutamine synthetase in leaves of *Nicotiana tabacum* L. by immunocytochemical methods. Plant Physiol.

[52] Milne RG, Lesemann DE (1984). Immunosorbent electron microscopy in plant virus studies. Methods Virol.

[53] Spurr AR (1969). A low-viscosity epoxy resin embedding medium for electron microscopy. J Ultrastruct Res.

