# Supplementary material for: Construction of a synthetic infectious cDNA clone of Grapevine Algerian latent virus (GALV-Nf) and its biological activity in Nicotiana benthamiana and grapevine plants
Source: Virol J. 2014 Nov 3;11:186. doi: 10.1186/1743-422X-11-186 (PMC4289286; doi:10.1186/1743-422X-11-186)
Supplement: Supplementary file 1 — Additional file 1: Table S1: Detection of GALV in different grapevine plants by RT-PCR. Survey of GALV in 152 grapevine samples from different geographic areas. (PDF 39 KB) [file 12985_2014_2511_MOESM1_ESM.pdf]

Sample	Specie	Location	Number of tested samples
**Arvino**	*V. vinifera*	Valle d'Aosta (IT)	20
**Dolcetto**	*V. vinifera*	Valle d'Aosta (IT)	3
**Fumin**	*V. vinifera*	Valle d'Aosta (IT)	1
**Petit Rouge**	*V. vinifera*	Valle d'Aosta (IT)	9
**Vien de Nus**	*V. vinifera*	Valle d'Aosta (IT)	3
**Barbera**	*V. vinifera*	Piemonte (IT)	1
**Bonarda**	*V. vinifera*	Piemonte (IT)	1
**Brachetto**	*V. vinifera*	Piemonte (IT)	2
**Cabernet Sauvignon**	*V. vinifera*	Piemonte (IT)	1
**Croatina**	*V. vinifera*	Piemonte (IT)	1
**Dolcetto**	*V. vinifera*	Piemonte (IT)	4
**Freisa**	*V. vinifera*	Piemonte (IT)	1
**Grignolino**	*V. vinifera*	Piemonte (IT)	1
**Lumassina**	*V. vinifera*	Piemonte (IT)	1
**Malvasia**	*V. vinifera*	Piemonte (IT)	1
**Moscato**	*V. vinifera*	Piemonte (IT)	4
**Nebbiolo**	*V. vinifera*	Piemonte (IT)	12
**Sangiovese**	*V. vinifera*	Piemonte (IT)	2
**Timorasso**	*V. vinifera*	Piemonte (IT)	1
**Vespolina**	*V. vinifera*	Piemonte (IT)	1
V. berlandieri	*V. berlandieri*	Piemonte (IT)	1
V. aestivalis	*V. aestivalis*	Piemonte (IT)	1
V. amurensis	*V. amurensis*	Piemonte (IT)	1
V. candicans	*V. candicans*	Piemonte (IT)	1
V. cinerea	*V. cinerea*	Piemonte (IT)	1
V. coignetia	*V. coignetia*	Piemonte (IT)	1
V. rupestris	*V. rupestris*	Piemonte (IT)	1
V. solonis	*V. solonis*	Piemonte (IT)	1
V. silvestris	*V. vinifera* L. subsp. *silvestris*	Piemonte (IT)	6
**3309**	*V. riparia* x *V. rupestris*	Piemonte (IT)	1
**101-14**	*V. riparia* x *V. rupestris*	Piemonte (IT)	1
**1103 P**	*V. berlandieri* x *V. rupestris*	Piemonte (IT)	1
**110R**	*V. berlandieri* x *V. rupestris*	Piemonte (IT)	1
**140 RU**	*V. berlandieri* x *V. rupestris*	Piemonte (IT)	1

Sample	Specie	Location	Number of tested samples
**161-49**	*V. berlandieri* x *V. rupestris*	Piemonte (IT)	1
**420A**	*V. berlandieri* x *V. rupestris*	Piemonte (IT)	1
**779P**	*V. berlandieri* x *V. rupestris*	Piemonte (IT)	1
**Cosmo 2**	*V. berlandieri* x *V. rupestris*	Piemonte (IT)	1
**Gravesac**	*V. riparia* x *V. rupestris*	Piemonte (IT)	1
**Kober 5BB**	*V. berlandieri* x *V. rupestris*	Piemonte (IT)	1
**Albarola**	*V. vinifera*	Liguria (IT)	1
**Bosco**	*V. vinifera*	Liguria (IT)	4
**Vermentino**	*V. vinifera*	Liguria (IT)	1
**Marzemino**	*V. vinifera*	Trentino (IT)	3
**Müller Thurgau**	*V. vinifera*	Trentino (IT)	6
**Pinot nero**	*V. vinifera*	Trentino (IT)	2
**Schiava grossa**	*V. vinifera*	Trentino (IT)	2
**101-14**	*V. riparia* x *V. rupestris*	Trentino (IT)	1
**110R**	*V. berlandieri* x *V. rupestris*	Trentino (IT)	1
**Arvino**	*V. vinifera*	Calabria (IT)	1
**Gaglioppo**	*V. vinifera*	Calabria (IT)	7
**Magliocco**	*V. vinifera*	Calabria (IT)	3
**Pecorello**	*V. vinifera*	Calabria (IT)	2
**1103 P**	*V. berlandieri* x *V. rupestris*	Marche (IT)	2
**420A**	*V. berlandieri* x *V. rupestris*	Marche (IT)	4
**Doukkali**	*V. vinifera*	Algeria	1
**Siegerrebe**	*V. vinifera*	Germany	1
**White muscat**	*V. vinifera*	Germany	1
**White muscat**	*V. vinifera*	Portugal	1
**Muscat of Alexandria**	*V. vinifera*	Portugal	1
**Beba**	*V. vinifera*	Portugal	1
**Muscat of Alexandria**	*V. vinifera*	Portugal	1
**Caracol**	*V. vinifera*	Portugal	1
**White muscat**	*V. vinifera*	Greece	1
**Incrocio Pirovano 1**	*V. vinifera*	Cyprus	1
**Lapithiotiko**	*V. vinifera*	Cyprus	1
**Muscat Susanna**	*V. vinifera*	Armenia	1
**Muskat Armyanski**	*V. vinifera*	Armenia	1
**Muscat Ottonel**	*V. vinifera*	Slovakia	1
**White muscat**	*V. vinifera*	Romania	1
**Rkatsiteli**	*V. vinifera*	France	2
